# Is team‐based perception of safety in the operating room associated with self‐reported wrong‐site surgery? An exploratory cross‐sectional survey among physicians

**DOI:** 10.1002/hsr2.42

**Published:** 2018-05-29

**Authors:** Stéphane Cullati, Delphine S. Courvoisier, Patricia Francis, Adriana Degiorgi, Paula Bezzola, Marc‐Joseph Licker, Pierre Chopard

**Affiliations:** ^1^ Quality of Care Division University Hospitals of Geneva Geneva Switzerland; ^2^ Department of General Internal Medicine, Rehabilitation and Geriatrics University of Geneva Geneva Switzerland; ^3^ Institute of Sociological Research University of Geneva Geneva Switzerland; ^4^ Quality of Care Unit Ente Ospedaliero Cantonale Lugano Switzerland; ^5^ Patient Safety Foundation Switzerland Zürich Switzerland; ^6^ Division of Anesthesiology University Hospitals of Geneva Geneva Switzerland

**Keywords:** operating room, perceptions, safety, wrong‐site surgery

## Abstract

**Aims:**

Participation in wrong‐site surgery may negatively influence the perception of safety by the health care professionals in the operating room (OR). The objective was to explore if perception of safety in the OR was seen as a team‐based or individualist concern and whether having participated in wrong‐site surgery was associated with perception of safety.

**Method and Results:**

Cross‐sectional survey at 2 annual meetings of surgery, in Switzerland, 2010. We used multivariate generalized models to assess the association of perception of safety in the OR (1 item) with self‐reported participation in wrong‐site surgery—overall, past (more than 3 y ago), or recent (last 3 y) participations—controlling for sociodemographic characteristics and opinion of the surgical safety checklist. One hundred ninety respondents answered the questionnaire (participation rate of 22.6%). Respondents mostly had a team‐based, rather than an individualistic, perception of safety in the OR. In multivariate analyses, the influence of ever participation in wrong‐site surgery was not significant. However, past participation in wrong‐site surgery (more than 3 y ago) was associated with perception of safety as team based, whereas recent participation (last 3 y) was associated—despite not significant at α ≤ 5%—with perception of safety as individualistic.

**Conclusion:**

In this sample, safety in the OR is most often seen as team based rather than individualistic. Perceiving safety in the OR as team based varies according to recent or past participation in wrong‐site surgery. Longitudinal research is needed to assess causality between participation in wrong‐site surgery and change in perception of safety.

## 1 INTRODUCTION

Safety culture is one important dimension of patient safety^1^ and reflects values, attitudes, and behaviors that health care professionals have in common when administering care to patients and includes their perception of safety as an individual and/or team‐based responsibility. Health care professionals are trained to be diligent in their care of patients and to follow the guiding principle “first do no harm.” Over the past decades, the development of a safety culture in health care—and, specifically, in the operating rooms (ORs)—has become a significant issue, and efforts have been made to enhance the importance of safety as a collective, rather than an individual, process.^2^ Yet changing attitudes towards safety perceptions has been difficult, mostly because of cultural reasons.^3^, ^4^ Furthermore, an excessive emphasis on individual responsibility can discourage team‐based safety procedures, such as the implementation of the surgical safety checklist.^5^

There has been significant progress in safety culture in health care settings over the past decade. The monitoring of safety indicators shows that health care professionals are more inclined to report medical errors, adverse events, or near‐miss errors.^6^ The worldwide implementation of the surgical safety checklist^5^ by the World Health Organization's initiative “Safe Surgery Saves Lives”^7^ and its frequent use by OR teams^8^, ^9^, ^10^, ^11^, ^12^ suggest that OR health care professionals have endorsed this team‐based safety procedure in their daily routine. However, in the OR, a persisting perception of safety uniquely based on individual competency may be problematic, because it can impede effective communication within the team and appropriate handling of team‐based safety procedures.^13^ Both individual and team‐based competencies are required to insure good safety of care in the OR. However, for historical reasons, many surgeons believe that OR safety is their responsibility. Currently, the prevalence of an individual‐based perception of safety in the OR is unknown.

Wrong‐site surgery is potentially devastating for the patient, but also for the health care professionals, who can become the “second victim” of these errors.^14^, ^15^, ^16^ Participation in errors (whether the health care professionals is responsible or not) can result in psychological regrets^17^ and mental health problems^18^, ^19^ and affect psychological well‐being.^20^ It may also negatively influence the perception of safety in the OR as a process that should be team based, but knowledge of this influence is limited. A study among otolaryngologists investigated the corrective actions to errors and adverse events and showed that actions included not only patients (error disclosure and mitigating the consequences of the errors) but also care practices within their units and departments, like time‐out, cross‐checking of patient identity, medications labelling, and other surgical protocols.^21^ Defensive changes, such as keeping the error for oneself, distancing, and escaping or avoidance, can affect clinical practice.^16^ However, it is unknown if defensive changes can modify safety attitude and, in particular, can affect the perception of safety as an individual responsibility. After participation in wrong‐site error surgery, the perception of colleagues can influence the feeling of guilt.^16^ The fears of loss trust and/or reputation by their colleagues and of making another error can change the attitude towards more individualistic safety attitudes. Conversely, constructive changes such as accepting responsibility, problem solving, seeking advices, learning from mistakes, and promoting changes could increase the perception of safety as a team‐based process. The impact of wrong‐site surgery on the “second victim” and how he/she responds is also influenced by the support provided by the health care organization in which the professional works. Unfortunately, this support is all too often lacking.^22^, ^23^

To evaluate the link between wrong‐site surgery participation and perception of safety of care in the OR as either an individual or team‐based process, we conducted an exploratory study using a self‐administered questionnaire distributed in 2 annual congresses of surgery, thereby allowing for its use in an anonymous and neutral environment. The primary objective of this exploratory study was to examine if safety in the OR was perceived as a team‐based process or as an individualist process. Secondarily, the objective was to assess the factors associated with the perception of safety in the OR and, in particular, to explore if self‐reported participation in wrong‐site surgery was associated with it. In other words, we tested the hypothesis that self‐reported participation in wrong‐site surgery is associated with a more individualistic perception of safety in the OR.

## 2 MATERIALS AND METHODS

### 2.1 Study design and settings

We conducted a cross‐sectional survey with a self‐administered questionnaire. The questionnaire—available in German, French, Italian, and English (see Appendix S1)—was distributed during 2 surgery meetings in Switzerland: the 97th Annual Meeting of the Swiss Society of Surgery,^24^ held in Interlaken, May 26‐28, 2010 (1100 to 1300 participants expected), and the 45th Annual Meeting of the European Society for Surgical Research,^25^ held in Geneva, June 9‐12, 2010 (300 to 350 participants expected). All congress participants were invited to fill out the questionnaires. In Interlaken, questionnaires were distributed during a single day. During the day of distribution, 7 bilingual medical students manually distributed questionnaires along with a short introductory letter. Participants who returned their questionnaire received a small Swiss chocolate. In Geneva, the questionnaire and the introductory letter were included within the materials package delivered to the participants at registration. The programs of both meetings were not related to wrong‐site surgery or to individual versus team‐based perceptions of safety in the OR. Translations of the questionnaire into German, English, and Italian were conducted by 2 native translators in each language and pretested in a sample of surgeons not participating in the surgery meetings. Pretest participants first filled the questionnaire and were then debriefed about each item in sequence, to identify problems (unclear questions, unfamiliar word, and appropriate answers). We stopped recruiting pretest participants when no new issues arose. This survey was exempted from ethic review by the Research Ethics Committee from the Geneva University Hospitals.

### 2.2 Dependent variable

Perception of safety as team based or individualistic in the OR was assessed by a single item: “Regarding safety of care in the operating theatre, to what extent are you in agreement with the following opinion: Safety is an individual concern above all, and a team concern to a lesser extent” (hereafter, *perception of safety*). Answers were given on a scale of 1 (do not agree at all) to 5 (fully agree). The variable was reverse coded to figure a high score for team‐based safety perception (and a low score of an individual‐based safety perception). To assess validity of this single item, we reported a convergent validity analysis (see Appendix S2).

### 2.3 Independent variable

#### 2.3.1 Self‐reported participation in wrong‐site surgery

The questions were as follows: “Have you participated in a surgical procedure where an operating site error (wrong side, level, procedure, or patient) took place with subsequent consequences for the patient (no matter who were the persons responsible): 1. during the last 3 years? 2. during your career?” The response modalities (yes, no) were distributed by type of error: level, side, procedure, and patient. If respondents answered “yes,” they were asked to indicate the number of errors. Then, we computed 2 types of variables: a dichotomous variable for participation in wrong‐site error (yes, no) and a count variable of the number of wrong‐site error participations. Both types of variables were coded (1) overall or (2) split into past participation (>3 y ago or before 2007) and recent participation (≤3 y ago or between 2007 and 2010). All of these variables were constructed irrespective of the type of errors.

### 2.4 Control variables

#### 2.4.1 Sociodemographic data

Information on sex, age, medical specialty (surgeons, anesthetist, others), clinical specialty (for surgeons only), work experience (number of years in clinical practice), working in private practice (yes, no), type of employment (university hospitals, nonuniversity public hospitals, private hospitals/clinics), annual operative load (number of interventions or procedures per year), and whether respondents had postgraduate medical training abroad (yes, no) was requested. Questions further assessed the type of institution where respondents performed their surgical procedures (university hospital, nonuniversity public hospital, private hospital/clinic).

#### 2.4.2 Opinion of the surgical safety checklist

Respondents answered questions about their perceptions of the surgical safety checklist (see Appendix S3). We calculated a global score (between 0 and 100, high score indicating positive perceptions) when at least 3 of the 8 items were answered (N = 179), after reverse coding 3 items (waste of time, no extra value, and efficacy). The opinion of the surgical safety checklist has been examined in more detail elsewhere.^26^

### 2.5 Statistical analysis

We examined univariable associations with perception of safety in the OR using analysis of variance when the independent variable was categorical and simple linear regression when the independent variable was continuous. Then, we examined 5 multivariate linear models: the first model examined factors of safety perception (objective 2) by including univariable associations significant at α ≤ 10% and one theoretically significant predictor (ie, number of year of clinical practice) (model 1). For inclusion of predictors, type I error was fixed at 10% to avoid missing important predictors. Then, we examined the influence of self‐reported participation in wrong‐site surgery on safety perception (objective 3) using different coding schemes: (*a*) a dichotomous variable of overall participation in wrong‐site surgery (model 2); (*b*) a 3‐category variable categorizing participation by periods of life: no participation, recent participation (last 3 y), and past (the rest of the career, ie, more than 3 y) participation in wrong‐site surgery (model 3); (*c*) a count variable of overall number of participation in wrong‐site surgery (model 4), 2 count variables separating between recent and past participations in wrong‐site surgery (model 5). To examine the influence of the period of participation in wrong‐site surgery (never, recent, and past), we excluded respondent having participated in both periods (models 3 and 5). All tests were 2 tailed, and significance level was set at 5%.

## 3 RESULTS

### 3.1 Sample description

A total of 843 questionnaires were distributed in both meetings (Interlaken 433 and Geneva 410), and 192 questionnaires were returned (128 in German, 40 in English, 23 in French, and 1 in Italian). After exclusion of respondents not working in a clinical field (researchers and managers), final number of respondents was 190 (participation rate of 22.6%).

Respondents were mostly men, surgeons, employed, without postgraduate training in another country, and with a median age of 42.5 years (range: 20‐79) (see Table 1). Most employed respondents worked in nonuniversity hospitals. Median number of years of practice was 14.0 years (range: 1‐40), and median annual number of interventions/procedures was 400 (mean: 707). Participants' characteristics were different between the 2 congresses, except postgraduate training in another country (data not shown). Overall, respondents had a positive opinion of the surgical safety checklist (median: 71.4).

**Table 1:** Sample characteristics of participants to the 2 surgery meetings,^a^ Switzerland, 2010

Characteristics	N (%)
Sex (N = 176)	
Men	119 (67.6)
Age [y] (N = 172)	
Mean (SD)	43.6 (11.9)
Median	42.5
Range	20‐79
By age groups	
<37	64 (37.2)
38‐50	62 (36.0)
51 and older	46 (26.7)
Profession (N = 175)	
Surgeons	119 (68.0)
Anesthetists	47 (26.9)
Others	9 (5.1)
Type of employment (N = 176)	
Private practitioners	21 (11.9)
Employed, working in (N = 154)	155 (88.1)
University hospital	47 (30.5)
Nonuniversity hospital	98 (63.6)
Private hospitals/clinics	9 (5.8)
Mean number of years of clinical practice (SD) (N = 143)	
Mean (SD)	14.8 (9.8)
Median	14.0
Median number of interventions/procedures by year (mean) (N = 133)	400 (707)
Having a postgraduate training in another country (N = 153)	54 (35.3)
Language of the questionnaire	
German	128 (67.4)
English	38 (20.0)
French	23 (12.1)
Italian	1 (0.5)
Mean (SD) opinion of the surgical safety checklist^b^ (N = 179)	67.9 (18.5)
Participation in wrong‐site surgery (N = 174)	
No	111 (63.8)
Yes	63 (36.2)
Number of participations in wrong‐site surgery (N = 163)	
0	111 (68.1)
1	23 (14.1)
2	15 (9.2)
3 or more	14 (8.6)
Periods of participation in wrong‐site surgery (N = 174)	
Never	111 (63.8)
Recent (last 3 y)	15 (8.6)
Past (more than 3 y ago)	34 (19.5)
Recent and past	14 (8.0)
Number of participations in wrong‐site surgery by periods (N = 158)^c^	
Recent only (last 3 y)	
0	145 (91.8)
1	8 (5.1)
2 or more	5 (3.2)
Past only (more than 3 y ago)	
0	126 (79.7)
1	15 (9.5)
2	9 (5.7)
3 or more	8 (5.1)

Abbreviation: SD, standard deviation

a 97th Annual Meeting of the Swiss Society of Surgery, Interlaken, May 26‐28, 2010, and 45th Annual Meeting of the European Society for Surgical Research, Geneva, June 9‐12, 2010.

b Between 0 and 100, high score indicating positive perception.

c Respondents having participated in wrong‐site surgery during both periods (past and recent) are excluded.

Participation in wrong‐site surgery (irrespective of the type of errors) was frequent, with 36.2% of respondents reporting at least one error over their whole practice (mean number of 0.7 errors) and 16.6% of respondents reporting a recent participation in wrong‐site surgery, ie, in the last 3 years (Table 1). Types of errors are reported in Appendix S4.

### 3.2 Perception of safety in the OR

Respondents mostly rejected the idea that “Safety is an individual concern above all, and a team concern to a lesser extent”: the mean score was 4.05 (SD: 1.19) out of 5—a high score indicating stronger rejection of safety as individualistic and the perception of safety in the OR to be *team based* instead.

In the univariable analysis, perception of safety as a team process was not different regardless of the respondents' characteristics (sex, age groups, profession, type of employment, number of year of clinical practice, and average number of interventions by year) (Table 2). However, individualistic (or lower score in the) perception of safety was most frequent among respondents working in university hospitals, having done postgraduate training in another country, and having filled the English version of the questionnaire. Having a positive opinion of the surgical safety checklist was associated with perception of safety as a team process, ie, a stronger rejection of safety as individualistic. Perception of safety was also significantly and positively associated with self‐reported participation in wrong‐site surgery (either the variable was treated as dichotomous or count): Wrong‐site surgery participation was associated more often with safety perception as a team process. Time of participation was also associated with perception of safety: Past participation was associated with stronger rejection of safety as individualistic compared with recent participation.

**Table 2:** Perception of safety in the operating room as a team process (univariable analysis)

Characteristics	Mean^a^	*P* Value^b^
Sex		.051
Women	4.31	
Men	3.93	
Age groups		.90
<37	4.10	
38‐50	4.02	
51 and older	4.00	
Profession		.24
Surgeon	4.03	
Anesthetist and others	4.26	
Type of employment		.99
Private practitioner	4.05	
Employed	4.05	
Working in		.001
University hospital	3.55	
Nonuniversity public hospital	4.30	
Private hospital/clinic	4.25	
Number of years of clinical practice	−0.013(0.010)^c^	.19
Number of interventions/procedures by year	0.000(0.000)^c^	.19
Postgraduate training in another country		.043
Yes	3.77	
No	4.18	
Language of the questionnaire		.001
German	4.28	
English	3.46	
French	3.74	
Opinion of the surgical safety checklist	0.015(0.05)^c^	.002
Participation in wrong‐site surgery		.037
Yes	3.90	
No	4.30	
Number of participations in wrong‐site surgery		
0	3.90	.045
1	4.65	
2	4.07	
3 or more	3.83	
Periods of participation in wrong‐site surgery		.011
Never	3.90	
Recent (last 3 y)	4.07	
Past (more than 3 y ago)	4.64	
Recent and past	3.69	
Number of participations in wrong‐site surgery by periods		
Recent (last 3 y) only		
0	4.07	.52
1	4.38	
2 or more	3.60	
Past (more than 3 y ago) only		
0	3.92	.037
1	4.80	
2	4.44	
3 or more	3.86	

a Mean can range between 1—synonym of individual‐based safety perception—and 5—team‐based safety perception.

b One‐way analysis of variance.

c Simple linear regression, with unstandardized coefficient (standard error).

In the multivariable analysis (Table 3), sex, number of years of clinical practice, working context, postgraduate training, and language of the questionnaire were not associated with perception of safety in the OR. However, opinion of the surgical safety checklist was positively associated in all models. When including overall participation in wrong‐site surgery, the association was not significant, either when the overall participation was treated as a dichotomous variable (model 2) or as a count variable (model 4). However, when categorizing wrong‐site surgery participation between recent (≤3 y) and past (>3 y), past participation was associated with increased safety perception as a team process, while recent participation was not. This result was similar when treating participation as a categorical variable (model 3) or as a count variable (model 5). Recent participation was not significant in models 3 and 5, but the effect size was in the opposite direction compared with past participation: Respondents having recently participated in wrong‐site surgery were less likely to report safety perception as a team process.

**Table 3:** Multivariable model of safety perceptions in the operating room as a team process^a^^,^ b

Characteristics	Difference in Mean Score (SE)				
	Without Self‐reported Participation in Wrong‐site Surgery (Model 1)	Adjusted with Self‐reported Participation in Wrong‐site Surgery		Adjusted with Self‐reported Number of Participation in Wrong‐site Surgery	
		Overall (Model 2)	Split between Recent and Past (Model 3)	Overall (Model 4)	Split between Recent and Past (Model 5)
Male sex (ref: male)	0.311(0.177)†	0.309(0.181)†	0.211(0.186)	0.291(0.190)	0.264(0.196)
Number of years of clinical practice	−0.016(0.014)	−0.017(0.014)	−0.021(0.014)	−0.017(0.015)	−0.023(0.015)
Working in (ref: university hospital)					
Private hospital/clinic	0.017(0.293)	0.083(0.299)	0.096(0.303)	0.041(0.294)	0.068(0.305)
Nonuniversity public hospital	0.532(0.296)†	0.573(0.298)†	0.540(0.294)†	0.514(0.280)†	0.535(0.304)†
Postgraduate training in another country (ref: no)	0.042(0.254)	0.063(0.259)	0.095(0.262)	0.060(0.272)	0.106(0.278)
Opinion of the surgical safety checklist	0.012(0.006)*	0.011(0.006)†	0.016(0.007)*	0.013(0.006)*	0.016(0.007)*
Overall participation in wrong‐site surgery (one or more errors) (ref: no)	…	0.092(0.214)	…	…	…
Period of participation in wrong‐site surgery (ref: never)^c^					
Recent (last 3 y)	…	…	−0.425(0.343)	…	…
Past (more than 3 y)	…	…	0.568(0.201)*	…	…
Number of overall participations in wrong‐site surgery	…	…	…	0.038(0.106)	…
Number of recent participations in wrong‐site surgery^b^	…	…	…	…	−0.228(0.246)
Number of past participations in wrong‐site surgery^b^	…	…	…	…	0.324(0.117)*
Number of respondents	97	95	86	91	84

Abbreviation: SE, standard error.

† *P* < .10.

* *P* < .05.

** *P* < .001.

a All models are adjusted with language of the questionnaire (German, English, and French), significant in univariable analysis.

b Single item “Regarding safety of care in the operating theatre, to what extent are you in agreement with the following opinion: Safety is an individual concern above all, and a team concern to a lesser extent” answers were given on a scale of 1 (do not agree at all) to 5 (fully agree), reverse coded to figure a high score synonym of team‐based safety perception (low score of an individual‐based safety perception).

c Respondents having participated in wrong‐site surgery during both periods (past and recent) are excluded.

In multivariable models stratified by medical professions (anesthesiologists vs surgeons), no significant association was observed between participation in wrong‐site surgery and perception of safety in the OR (Appendix S5). However, the main finding that past participation in wrong‐site surgery was associated with safety perception as a team process was mostly supported among anesthesiologists.

## 4 DISCUSSION

The results of this exploratory study showed that most respondents endorsed a team‐based perception of safety in the OR, rather than an individualistic perception. All factors negatively associated in the univariable analyses with a perception of safety (working in university hospitals, having a postgraduate training in another country, and filling the English version of the questionnaire) became not significant in the multivariate analyses, except for opinion of the surgical safety checklist, which was positively associated (perception of safety as a team process increased when respondents had a positive perception of the surgical safety checklist). Two interpretations could be made: First, the absence of subgroup differences suggests that a team‐based perception of safety may be generally widespread among surgeons and anesthetists, irrespective of their clinical experience, working context, and sociodemographic characteristics; second, there is a possibility of missing true associations (type II error) due to lack of power in multivariate analyses. Therefore, this result can tentatively be interpreted as good news for the development of safety culture but should be taken with caution as the study was conducted in a selected population of physicians and the participation rate was low, which could raise concerns about selection bias. Additional research should be conducted to confirm this result.

This study examined the association of perception of safety in the OR with self‐reported participation in wrong‐site surgery. Some publications have assessed safety attitudes in the OR,^27^, ^28^, ^29^ but none have addressed the association between safety perception and wrong‐site surgery. To our knowledge, this is a novel study. Our results showed that participation in itself (yes versus no), or the number of participation in wrong‐site surgery during the whole clinical practice, was not related to perception of safety in the OR. However, when splitting participation in wrong‐site surgery according to time of participation (recent versus past participation), we found a differential impact: For recent participation, the effect was negatively—but not significantly—associated with perception of safety, signifying that recent experience of wrong‐site surgery favors individualistic perception of safety. In contrast, past participation (more than 3 y ago) was significantly positively associated, ie, favored a team‐based perception of safety. This result was similar when treating participation in wrong‐site surgery as a dichotomous variable (model 3) or a count variable (model 5). To our knowledge, this is a novel finding and could be interpreted in terms of psychological coping processes with errors.^30^ Doctors' interpretation of the error may evolve over time. When the error is recent, doctors may think in terms of individual responsibilities and, potentially, legal issues. Emotionally, wrong‐site surgery—like medical errors in general—could be psychologically devastating for doctors^18^, ^19^, ^23^, ^31^, ^32^, ^33^ and influence their perception of safety in general^34^ or in the OR. One emotional reaction could be a hyperindividualistic behavior in terms of safety within the OR: The doctor would want to control everything himself or herself, without delegating responsibilities to other team members. This effect could be reinforced by a lack of social support among doctors hindering free discussion of errors with peers^17^, ^19^, ^32^, ^35^, ^36^ and a lack of support of the hospital or perceived barriers to seeking counselling.^23^ In contrast, when the error was committed more than 3 years ago, it could evolve into positive and rational behavioral changes. Emotional reactions and potential fears of legal issues could have diminished with time, and root cause investigations could have shown other systemic causes. Further research is needed to replicate this finding of an evolution of the impact of wrong‐site surgery but also to determine when this evolution occurs. A longitudinal survey would be necessary to examine how the impact of wrong‐site surgery evolves over time. The cross‐sectional nature of our data does not allow such examination and calls for caution with this interpretation of our findings.

In analyses stratified by medical professions (anesthesiologists vs surgeons), despite the fact that no association remained significant, the comparison of the effect sizes directions suggested that participation in wrong‐site surgery was linked to a more team‐based safety perception mostly among anesthesiologists. This result, very preliminary and based on a simple comparison of effect sizes, is interesting, but we cannot conclude on it because of a lack of statistical power. Potentially, one explanation could be that anesthesiologists often receive more training on teamwork for clinical safety than surgeons.

The time lag of 3 years after the error was committed used in this study was arbitrary and did not relate to any natural or psychological process occurring over time. We did not ask the date of the wrong‐site surgery events, as we thought this information was too sensitive. Therefore, it was not possible to determine when the effect reversed. We simply asked respondents to distinguish between errors that occurred within the last 3 years and prior.

Our results also showed that the number of overall participation in wrong‐site surgery was not associated with perception of safety in the OR. Three possible interpretations could be put forward. First, the number of self‐reported participation may be too small and the present study may have missed the association. Second, we can hypothesize that doctors that have had multiple participations may have used psychological coping strategies like medical errors acceptance,^17^, ^37^ in which doctors think that medical errors are expected and known to occur during routine care and, therefore, perceive the learning process of errors as futile. Third, we do not know if the participants who declared wrong‐site surgery were directly involved in this or not (ie, responsible), as the direct responsibility of the respondent was not questioned. Possibly, most respondents reported participation in which they were not directly implicated or responsible, which could explain this lack of association.

### 4.1 Limitations and strengths

Nine points have to be considered. First, the participation rate was low (22.6%), rising concerns about selection bias in the prevalence of team safety perception. Low participation rates are not uncommon when surveying doctors^38^, ^39^ and are not automatically synonymous with nonresponse bias^40^ and do not systematically imply an important bias.^41^, ^42^ Second, this cross‐sectional study was not designed to be representative of professions working in the OR. Consequently, its findings cannot be generalized. Third, because of the cross‐sectional design of this survey, we cannot determine the causality of the association between perception of safety in the OR and self‐reported participation in wrong‐site surgery. It is worth noting that the reverse association—higher individualistic perceptions of safety in the OR are associated with increased number of self‐reported participation in wrong‐site surgery—is theoretically sound and plausible and may be examined in future studies.^43^ Fourth, this study may be affected by information bias. Wrong‐site surgery is a sensitive topic and many respondents may have underreported their errors. Fifth, we compiled data from 2 congresses of surgery into one sample. We replicated the multivariate models in the sample of Interlaken only and found no differences with the overall sample (data not shown), with the exception of opinion of the surgical safety checklist, which was not significantly associated with perception of safety in all 5 models. Sixth, we assessed safety culture in the OR by administering a survey in 2 surgical congresses. Thus, it limits our capacity to understand safety culture as an interprofessional concept. Moreover, past studies have shown differences in safety perceptions between physicians and nurses.^44^, ^45^, ^46^, ^47^ Further research will be needed to replicate this survey among nonphysicians professions working in the OR, such as scrub nurses, anesthetist nurses, auxiliary nurses, or other professionals involved in OR safety. Seventh, we cannot avoid the possibility of duplicate responses between the 2 congresses, although we consider this risk to be reasonably low because the congresses were organized by different surgical societies and in different cities. Eighth, the data collection of this study was conducted in 2010. Considering both evolving^48^ and persistent^49^ trends in the safety culture of acute health care institutions in high‐income countries over the recent decades, the relevance of these findings need to be updated. Ninth, an analysis of convergent validity (Appendix S2) showed that the outcome, perception of safety in the OR, was weakly correlated with other items or scales related to safety. Despite suggesting weak association with similar concepts, this result is also in line with reviews suggesting that the concept of safety (safety culture and safety climate), imported from other industries, varies considerably across scales (high heterogeneity of face validity),^50^ and theoretical underpinnings are often lacking.^51^

## 5 CONCLUSION

Respondents mostly had a team‐based perception of safety in the OR. Safety in the OR was perceived differently (team based or individualistic) depending on respondents having had past or recent participation in wrong‐site surgery. Further research should be conducted using random and larger samples to confirm these results and to determine if and when the reversal impact of wrong‐site surgery occurs on perception of safety in the OR.

## FUNDING

No source of funding.

## CONFLICT OF INTEREST

The authors declare having no conflict of interest.

## AUTHOR CONTRIBUTIONS

Conceptualization: Stéphane Cullati, Delphine S. Courvoisier, Patricia Francis, Adriana Degiorgi, Paula Bezzola, Marc‐Joseph Licker, Pierre Chopard

Data curation: Stéphane Cullati

Formal analysis: Stéphane Cullati, Delphine S. Courvoisier

Investigation: Stéphane Cullati, Marc‐Joseph Licker

Methodology: Stéphane Cullati, Marc‐Joseph Licker, Pierre Chopard, Patricia Francis

Project administration: Stéphane Cullati, Pierre Chopard

Software: Stéphane Cullati

Supervision: Marc‐Joseph Licker, Pierre Chopard

Validation: Stéphane Cullati, Delphine S. Courvoisier, Patricia Francis, Adriana Degiorgi, Paula Bezzola, Marc‐Joseph Licker, Pierre Chopard

Writing – original draft: Stéphane Cullati

Writing – review and editing: Stéphane Cullati, Delphine S. Courvoisier, Patricia Francis, Adriana Degiorgi, Paula Bezzola, Marc‐Joseph Licker, Pierre Chopard

## Supporting information

Appendix 1Questionnaire
Appendix 2Convergent validity of the dependent variable
Appendix 3Perception of the Surgical Safety Checklist
Appendix 4Number of self‐reported wrong‐site surgery, by type of error
Appendix 5Multivariable model of safety perceptions in the operating room as a team process, stratified by medical professions.
