# Supplementary material for: Is team‐based perception of safety in the operating room associated with self‐reported wrong‐site surgery? An exploratory cross‐sectional survey among physicians
Source: Health Sci Rep. 2018 May 29;1(6):e42. doi: 10.1002/hsr2.42 (PMC6266346; doi:10.1002/hsr2.42)
Supplement: Supplementary file 1 — Appendix 1Questionnaire Appendix 2Convergent validity of the dependent variable Appendix 3Perception of the Surgical Safety Checklist Appendix 4Number of self‐reported wrong‐site surgery, by type of error Appendix 5Multivariable model of safety perceptions in the operating room as a team process, stratified by medical professions. [file HSR2-1-e42-s001.docx]

# Supplementary Materials

**Appendix 1 Questionnaire**

**Appendix 2 Convergent validity of the dependent variable**

**Appendix 3 Perception of the Surgical Safety Checklist**

**Appendix 4 Number of self-reported wrong-site surgery, by type of error**

**Appendix 5 Multivariable model of safety perceptions in the operating room as a team process, stratified by medical professions.**

**The surgical safety checklist in your healthcare institution**

1) The institution in which you perform your surgical procedures is a … (*please tick one box only*)

❑ university hospital ❑ non-university public hospital ❑ private hospital/clinic

2) Has a surgical safety checklist similar to the one proposed by the World Health Organization been implemented in your hospital/clinic for procedures carried out in operating theatres?

1. Yes ❑ ☞ *go directly to question* ***3***

2. No ❑ ☞ *go directly to question* ***6***

3. Don’t know ❑ ☞ *go directly to question* ***6***

4. I don’t know this surgical safety checklist ❑ ☞ *go directly to question* ***7***

3) In which year was the checklist introduced?

4) In your opinion, to what extent was the implementation of the checklist supported by…

(*please tick the corresponding box for each line*) No Medium Very strong No

support support support opinion

1. Your hospital/clinic senior management 1 2 3 4 5 ❑
2. The department of surgery senior management 1 2 3 4 5 ❑
3. The department of anaesthesiology senior management 1 2 3 4 5 ❑
4. The nursing department (including instrumentists) senior management 1 2 3 4 5 ❑

5) This checklist is comprised of the following sections: (*please tick the corresponding box for each line*)

No Yes

1. Sign In (before induction of anaesthesia) ❑ ❑ ☞
2. Time Out (before skin incision) ❑ ❑ ☞
3. Sign Out (after the procedure) ❑ ❑ ☞
4. Other section/s; please mention: ❑ ❑ ☞

☞ For each box ticked "yes", please indicate if these checklist sections are applied either never, rarely, partially, very often, or quasi systematically within *your surgical/anaesthetic team* (i.e., the one with which you operate most often):

Never Rarely Partially Very often Quasi systematically

(0%) (1-29%) (30-59%) (60-90%) (>90%)

1. Sign In ❑ ❑ ❑ ❑ ❑

2. Time Out ❑ ❑ ❑ ❑ ❑

3. Sign Out ❑ ❑ ❑ ❑ ❑

4. Other ❑ ❑ ❑ ❑ ❑

**Your opinion of the checklist**

6) Regarding the use of the checklist in operating theatres, to what extent are you in agreement with the following opinions: *(please circle a number for each statement)*

*The checklist…* Don't agree at all Partially Fully agree

agree No opinion

1. improves the safety of procedures (anaesthetic and surgical) 1 2 3 4 5 ❑
2. is a waste of time 1 2 3 4 5 ❑
3. improves team communication (related to safety) 1 2 3 4 5 ❑
4. brings no extra value to *existing* safety procedures already in place in my

hospital/clinic *before* its implementation 1 2 3 4 5 ❑

1. helps to develop a safety culture in surgical teams 1 2 3 4 5 ❑
2. has not demonstrated its efficacy in the scientific literature 1 2 3 4 5 ❑
3. facilitates teamwork 1 2 3 4 5 ❑
4. eliminates (during the controls) the hierarchy between healthcare professionals

(doctors, nurses, etc.) 1 2 3 4 5 ❑

1. If you wish to express another opinion, please mention below:

**Your opinion on safety culture**

☞ Please turn the page

7) If you think about *your anaesthetic/surgical team*, to what extent are you in agreement with the following opinions: *(please circle a number for each statement)*

Don’t agree at all Partially Fully agree

agree No opinion

1. Staff routinely discusses ways to prevent incidents from happening again 1 2 3 4 5 ❑
2. The culture is one of continuous improvement 1 2 3 4 5 ❑
3. The team has a shared understanding and vision about safety issues; everyone is

equally valued and feels free to contribute 1 2 3 4 5 ❑

1. Staff feel free to question the decisions or actions of those with more authority 1 2 3 4 5 ❑
2. There is a blame culture, so staff are reluctant to report incidents 1 2 3 4 5 ❑
3. If you wish to express another opinion, please mention below:

8) Regarding safety of care in the operating theatre, to what extent are you in agreement with the following opinions: *(please circle a number for each statement)*

Don’t agree at all Partially Fully agree

agree No opinion

1. Safety is an individual concern above all, and a team concern to a lesser extent 1 2 3 4 5 ❑
2. Safety is dependent not only on the responsibility of doctors, but of all healthcare

professionals (nurses, auxiliary-nurses, etc) 1 2 3 4 5 ❑

1. If you wish to express another opinion, please mention below:

**Wrong site surgery**

9) Have you participated in a surgical procedure where an operating site error (wrong side, level, procedure, or patient) took place with subsequent consequences for the patient (*no matter who were the persons responsible*) during the last 3 years? during your career?

**During your career**

**Between 2007-2009 (until end 2006)**

No Yes How many? No Yes How many?

*An error …*

1. wrong side ❑ ❑ ❑ ❑
2. wrong level ❑ ❑ ❑ ❑
3. wrong procedure ❑ ❑ ❑ ❑
4. wrong patient ❑ ❑ ❑ ❑

**Some personal data**

10) Are you… ❑ male ❑ female

11) Your date of birth (*year only*):

12) You are … ❑ surgeon ☞ ❑ anaesthetist ☞ ❑ nurse ❑ other (define):

☞ The number of years you have been practising in surgery/anaesthetics:

13) The average number of interventions/procedures that you perform each year:

14) Are you… ❑ private practitioner (consulting rooms, either alone or with colleagues)

❑ working in a ☞ ❑ university hospital

❑ non-university public hospital

❑ private hospital/clinic

15) In which country are you working?

16) After completing your specialist training, have you followed part of your postgraduate training in another country? ❑ No ❑ Yes, in which country? ❑ Not applicable

**Thank you for your valuable collaboration !**

**Appendix 2 Convergent validity of the dependent variable^1^ with available items and scale in the questionnaire**

| **Scale** | **Items** | **Expected correlation** | **Observed correlation*** | **p-value**** |
| --- | --- | --- | --- | --- |
| (No) | “Safety is dependent not only on the responsibility of doctors, but of all healthcare professionals (nurses, auxiliary-nurses, etc)”  *[response options: 1 (don’t agree at all) to 5 (fully agree)]* | Positive coefficient: the higher the safety implies all professional, the more team-based is safety. | 0.221 | 0.006 |
| Opinion of the surgical safety checklist | See article and appendix 3 | Positive coefficient: the more positive the opinion toward the surgical safety checklist, the more team-based is safety | 0.276 | <0.001 |
| Attitudes toward safety culture (5 items selected and adapted from the Pharmacy Safety Climate Questionnaire, reference: ([52](#_ENREF_52))) | 1. Staff routinely discuss ways to prevent incidents from happening again.  2. The culture is one of continuous improvement.  3. The team has a shared understanding and vision about safety issues; everyone is equally valued and feels free to contribute.  4. Staff feel free to question the decisions or actions of those with more authority.  5. There is a blame culture, so staff are reluctant to report incidents.  *[response options: 1 (don’t agree at all) to 5 (fully agree)]*  *A score based on item 1 to 4 had the following performance:*  *- Cronbach alpha 0.84 ;*  *- 1 latent variable (factor) based on principal component analysis, explaining 67% of the variance* | Positive coefficient: the higher the attitudes toward safety culture, the more team-based is safety | -0.089 | 0.322 |

^1^ perception of safety in the OR, measured with the single item: “Regarding safety of care in the operating theatre, to what extent are you in agreement with the following opinion: Safety is an individual concern above all, and a team concern to a lesser extent.” Answers were given on a scale of 1 (don’t agree at all) to 5 (fully agree).

* Pearson correlation or Spearman’s rho.

** Two-tailed and significance level at 5%

**Appendix 3**

**Perceptions of the Safety Surgical Checklist**

| *Regarding the use of the checklist in operating theatres, to what extent are you in agreement with the following opinions:* | Don’t agree at all or don’t agree | Partially agree | Fully agree or agree | Mean Score* (SD) |
| --- | --- | --- | --- | --- |
| *The checklist…* | N(%) | N(%) | N(%) |  |
| improves the safety of procedures (anaesthetic and surgical) (N=178) | 8(4.5) | 12(6.7) | 158(88.8) | 4.5 (0.9) |
| is a waste of time (N=173) | 122(70.5) | 24(13.9) | 27(15.6) | 2.0 (1.3) |
| improves team communication (related to safety) (N=175) | 24(13.7) | 31(17.7) | 120(68.6) | 3.8 (1.1) |
| brings no extra value to *existing* safety procedures already in place in my hospital/clinic *before* its implementation (N=164) | 95(57.9) | 36(22.0) | 33(20.1) | 2.4 (1.3) |
| helps to develop a safety culture in surgical teams (N=173) | 19(11.0) | 23(13.3) | 131(75.7) | 4.0 (1.1) |
| has not demonstrated its efficacy in the scientific literature (N=126) | 35(20.6) | 55(32.4) | 80(47.1) | 2.5 (1.3) |
| facilitates teamwork (N=170) | 35(20.6) | 55(32.4) | 80(47.1) | 3.4 (1.1) |
| eliminates (during the controls) the hierarchy between healthcare professionals (N=161) | 69(42.9) | 49(30.4) | 43(26.7) | 2.8 (1.3) |

* Don’t agree at all= 1, Don’t agree=2, Partially agree=3, Agree=4, Fully agree=5

**Appendix 4 Number of self-reported wrong-site surgery, by type of error.**

|  | Errors occurring | | | | Errors during  all career until 2009  (A or B) | |
| --- | --- | --- | --- | --- | --- | --- |
|  | between 2007-2009  (A) | | during the career, until end 2006  (B) | |  |  |
|  | N (%) | *If yes, how many*:  Mean (SD),  range | N (%) | *If yes, how many*:  Mean (SD),  range | N (%) | *If yes, how many*:  Mean (SD),  range |
| Type of error: |  |  |  |  |  |  |
| side | 16 (9.2) | 1.3 (0.6),  1-3 | 38 (21.8) | 1.6 (1.0),  1-5 | 49 (28.2) | 1.6 (1.2),  1-6 |
| level | 5 (2.9) | 1.4 (0.6),  1-2 | 12 (6.9) | 1.5 (0.8),  1-3 | 15 (8.6) | 1.6 (0.7),  1-3 |
| procedure | 9 (5.2) | 1.1 (0.4),  1-2 | 19 (10.9) | 2.0 (1.5),  1-5 | 22 (12.6) | 2.2 (1.7),  1-6 |
| patient | 8 (4.6) | 1.3 (0.5),  1-2 | 16 (9.2) | 1.3 (0.5),  1-2 | 20 (11.5) | 1.5 (0.7),  1-3 |

**Appendix 5 Multivariable model of safety perceptions in the operating room as a team process^a^, stratified by medical professions.**

|  | **anesthesiologists** | | | | | **surgeons** | | | | |
| --- | --- | --- | --- | --- | --- | --- | --- | --- | --- | --- |
|  | Safety in the OR^a^ | adjusted with self-reported participation in wrong-site surgery | | adjusted with self-reported number of participation in wrong-site surgery | | Safety in the OR^a^ | adjusted with self-reported participation in wrong-site surgery | | adjusted with self-reported number of participation in wrong-site surgery | |
|  | model 1 | Overall  (model 2) | Split between recent and past  (model 3) | Overall  (model 4) | Split between recent and past  (model 5) | model 1 | Overall  (model 2) | Split between recent and past  (model 3) | Overall  (model 4) | Split between recent and past  (model 5) |
|  | B(SE) | B(SE) | B(SE) | B(SE) | B(SE) | B(SE) | B(SE) | B(SE) | B(SE) | B(SE) |
| Male sex (ref: male) | 0.12(0.34) | 0.21(0.36) | 0.07(0.31) | -0.10(0.38) | -0.15(0.31) | 0.37(0.23) | 0.43(0.24)† | 0.27(0.27) | 0.45(0.24)† | 0.28(0.27) |
| Number of years of clinical practice | 0.01(0.03) | 0.03(0.04) | 0.02(0.03) | 0.06(0.03) | 0.01(0.04) | -0.03(0.18) | -0.03(0.02) | -0.03(0.02) | -0.03(0.02) | -0.03(0.02)† |
| Working in (ref: university hospital) |  |  |  |  |  |  |  |  |  |  |
| private hospital / clinic | 0.03(0.57) | 0.32(0.55) | 0.47(0.88) | 0.30(0.71) | 0.47(0.96) | 0.08(0.39) | 0.17(0.40) | 0.12(0.40) | 0.21(0.41) | 0.14(0.40) |
| non-university public hospital | 0.79(0.57) | 0.80(0.51) | 0.74(0.79) | 0.69(0.72) | 0.78(0.97) | 0.47(0.33) | 0.57(0.33)† | 0.57(0.33)† | 0.61(0.34)† | 0.60(0.34)† |
| Postgraduate training in another country (ref: no) | -0.09(0.44) | -0.35(0.54) | -0.31(0.49) | -0.22(0.51) | -0.27(0.49) | 0.10(0.33) | 0.24(0.36) | 0.19(0.38) | 0.28(0.36) | 0.19(0.38) |
| Opinion of the Surgical Safety Checklist | -0.00(0.01) | 0.00(0.01) | 0.02(0.01) | 0.01(0.01) | 0.01(0.01) | 0.01(0.01)† | 0.01(0.01)† | 0.01(0.01)† | 0.01(0.01)† | 0.01(0.01) |
| Overall participation in wrong-site surgery (one or more errors) (ref: no) | - | 0.53(0.49) | - | - | - | - | -0.26(0.30) | - | - | - |
| Period of participation in wrong-site surgery (ref: never)^b^ |  |  |  |  |  |  |  |  |  |  |
| recent (last 3 years) | - | - | 0.10(0.73) | - | - | - | - | -0.57(0.35) | - | - |
| past (more than 3 years) | - | - | 0.72(0.55) | - | - | - | - | 0.44(0.39) | - | - |
| Numbers of overall participations in wrong-site surgery | - | - | - | 0.12(0.23) | - | - | - | - | -0.21(0.18) | - |
| Numbers of recent participations in wrong-site surgery^a^ | - | - | - | - | 0.28(0.48) | - | - | - | - | -0.39(0.26) |
| Numbers of past participations in wrong-site surgery^a^ | - | - | - | - | 0.17(0.21) | - | - | - | - | 0.45(0.28) |
| Number of respondents | 28 | 28 | 23 | 25 | 22 | 68 | 66 | 62 | 65 | 61 |
| All models are adjusted with language of the questionnaire (German, English, French), significant in univariable analysis.  OR= Operating Room  † p<0.10,*p<0.05, **p<0.001  ^a^ single item “Regarding safety of care in the operating theatre, to what extent are you in agreement with the following opinion: Safety is an individual concern above all, and a team concern to a lesser extent”, answers were given on a scale of 1 (don’t agree at all) to 5 (fully agree), reverse coded to figure a high score synonym of team based safety perception (low score of an individual based safety perception).  ^b^ respondents having participated in wrong-site surgery during both periods (past and recent) are excluded. | | | | | | | | | | |
